# Circulating Neutrophil Extracellular Traps Signature for Identifying Organ Involvement and Response to Glucocorticoid in Adult-Onset Still’s Disease: A Machine Learning Study

**DOI:** 10.3389/fimmu.2020.563335

**Published:** 2020-11-09

**Authors:** Jinchao Jia, Mengyan Wang, Yuning Ma, Jialin Teng, Hui Shi, Honglei Liu, Yue Sun, Yutong Su, Jianfen Meng, Huihui Chi, Xia Chen, Xiaobing Cheng, Junna Ye, Tingting Liu, Zhihong Wang, Liyan Wan, Zhuochao Zhou, Fan Wang, Chengde Yang, Qiongyi Hu

**Affiliations:** ^1^ Department of Rheumatology and Immunology, Ruijin Hospital, Shanghai Jiao Tong University School of Medicine, Shanghai, China; ^2^ Department of Rheumatology and Immunology, The First People’s Hospital of Yancheng, The Fourth Affiliated Hospital of Nantong University, Yancheng, China

**Keywords:** adult-onset Still’s disease, circulating neutrophil extracellular traps, organ involvement, response to glucocorticoid, machine learning

## Abstract

Adult-onset Still’s disease (AOSD) is an autoinflammatory disease with multisystem involvement. Early identification of patients with severe complications and those refractory to glucocorticoid is crucial to improve therapeutic strategy in AOSD. Exaggerated neutrophil activation and enhanced formation of neutrophil extracellular traps (NETs) in patients with AOSD were found to be closely associated with etiopathogenesis. In this study, we aim to investigate, to our knowledge for the first time, the clinical value of circulating NETs by machine learning to distinguish AOSD patients with organ involvement and refractory to glucocorticoid. Plasma samples were used to measure cell-free DNA, NE-DNA, MPO-DNA, and citH3-DNA complexes from training and validation sets. The training set included 40 AOSD patients and 24 healthy controls (HCs), and the validation set included 26 AOSD patients and 16 HCs. Support vector machines (SVM) were used for modeling and validation of circulating NETs signature for the diagnosis of AOSD and identifying patients refractory to low-dose glucocorticoid treatment. The training set was used to build a model, and the validation set was used to test the predictive capacity of the model. A total of four circulating NETs showed similar trends in different individuals and could distinguish patients with AOSD from HCs by SVM (AUC value: 0.88). Circulating NETs in plasma were closely correlated with systemic score, laboratory tests, and cytokines. Moreover, circulating NETs had the potential to distinguish patients with liver and cardiopulmonary system involvement. Furthermore, the AUC value of combined NETs to identify patients who were refractory to low-dose glucocorticoid was 0.917. In conclusion, circulating NETs signature provide added clinical value in monitoring AOSD patients. It may provide evidence to predict who is prone to be refractory to low-dose glucocorticoid and help to make efficient therapeutic strategy.

## Introduction

Adult-onset Still’s disease (AOSD) is a systemic autoinflammatory disease, typically characterized by fever of unknown origin, evanescent rash, polyarthralgia, and even life-threatening complications, such as macrophage activation syndrome (MAS), fulminant hepatitis, and cardiopulmonary system involvement (1). Cardiopulmonary involvement was defined as pericarditis, pneumonia, pleuritic, and pulmonary arterial hypertension (PAH) as well as any cardiac or pulmonary disorder related to the AOSD (2). Due to the non-specific clinical and laboratory features, the diagnosis of AOSD often necessitates the exclusion of infectious, neoplastic, and autoimmune disease, leaving a challenge in practice (3). In our previous study of 61 AOSD patients, we reported that some patients exhibited an unfavorable outcome, and 6 patients died of severe pneumonia, liver failure, and MAS (4). Therefore, identification of predictive models to distinguish who is prone to develop life-threatening complications is crucial to improve the efficacy of treatment in AOSD.

Due to the complexity of the disease characteristics, therapy of AOSD remains empirical (5). Glucocorticoid therapy is considered as the first-line therapy of AOSD, whereas low-dose prednisone is not effective in controlling the disease in some patients. Intravenous infusion of high-dose methylprednisolone, use of disease modifying antirheumatic drugs (DMARDs), and several biologic agents are essential for refractory AOSD or patients with severe complications (6). For this reason, how to balance the side effects and effectiveness is of great clinical significance in the therapy of AOSD. Thus, identification of biomarkers to predict treatment response is also an urgent need.

To date, the pathogenesis of AOSD is mostly hypothetical, but a generally growing understanding of AOSD suggest the involvement of neutrophils (7). More than 80% of AOSD patients present with neutrophilic leukocytosis during the acute flare (2). Histology of the rash, lymph nodes, and liver are also characterized by neutrophil infiltration (8), suggesting that neutrophils are critical effector cells in AOSD.

Along with releasing various granule proteins, neutrophils also release neutrophil extracellular traps (NETs), which have been shown to play a role in immune-mediated conditions (9). NETs can mediate sterile tissue damage and promote inflammatory responses, including systemic lupus erythematosus (SLE), rheumatoid arthritis (RA), and vasculitis (10). In view of the crucial role of neutrophils in AOSD pathogenesis, much attention has been paid to the role of NETs, a new way of neutrophil death (11). Our previous study has demonstrated a greater ability of neutrophils from AOSD patients to form NETs, which could induce inflammatory macrophages by activating NLR family, pyrin domain containing 3 (NLRP3) inflammasome, and stimulating cytokine production (12). Upon citrullinated, citrullinated histone 3 (citH3) in neutrophils contributes to chromatin decondensation and nuclear membrane disruption. Granule proteins, including myeloperoxidase (MPO) and neutrophil elastase (NE), are involved in the breakdown of membranaceous structures, allowing for mixture of nuclear and cytoplasmic contents. Finally, cell-free DNA (cfDNA) and granule proteins are released as NETs (13). Recent studies have indicated that circulating NETs are potential biomarkers in many rheumatic diseases (14). In SLE patients, circulating levels of NETs could identify patients with active disease and severe disease, including kidney involvement and cardiovascular disease (15, 16). MPO-DNA, a circulating NET complex, was found to be positively associated with rheumatoid factor (RF) levels, anti-citrullinated protein/peptide antibodies (ACPA) titers, and neutrophil counts in patients with RA (17). In view of the diagnostic role of circulating NETs, it may shed new light on the identification of a novel diagnostic model in AOSD.

Previous studies focus on the pro-inflammatory and tissue-damaging effects of NETs, while the associations between circulating NETs and clinical manifestations, especially important organ involvement and treatment response in AOSD, are still undetermined. NETs are the complex of various granule proteins, so we hypothesize that a multidimensional feature model of NETs will result in good prediction and evaluation. In this study, a total of four circulating NETs, including citH3-DNA, NE-DNA, MPO-DNA, and cfDNA, were investigated in patients with AOSD to evaluate the relationship of circulating NETs with disease activity, clinical parameters, organ involvement, and treatment response to glucocorticoid.

## Methods

### Patients and Healthy Subjects

The study population consisted of 66 AOSD patients fulfilling Yamaguchi’s criteria after exclusion of those with infectious, neoplastic, and autoimmune disorders. Information on demographic and clinical data was entered into a database together with the laboratory test results. The training set included 40 AOSD and 24 healthy controls (HCs). An independent set of 26 AOSD and 16 HCs was included as a validation set. The systemic disease activity of each AOSD patient was assessed using a modified Pouchot’s score (18). The study was performed in accordance with the Declaration of Helsinki and the principles of Good Clinical Practice. Biological samples were obtained under a protocol approved by the Institutional Research Ethics Committee of Ruijin Hospital (ID: 2016–62), Shanghai, China. All subjects signed written informed consent.

### Quantification of Cell-Free DNA and NET-DNA Complexes in the Serum of AOSD Patients

Cell-free DNA was quantified in serum using the Quant-iT PicoGreen double-stranded DNA (dsDNA) assay kit (Invitrogen, USA) according to the manufacturer’s instructions. Ten percent serum was added per well, followed by incubation for 10 min away from light. NE-DNA, MPO-DNA, and citH3-DNA complexes were quantified using the Quant-iT PicoGreen as previously described (19). As the capturing antibody, anti-citH3, NE, and MPO monoclonal antibody (Abcam, Serotec, USA) was coated onto 96-well microtiter plates overnight at 4°C. After blocking in 1% BSA for 90 min at room temperature, 10% serum was added per well, followed by incubation overnight at 4°C. The plate was washed 5 times, followed by the addition of PicoGreen from the kit described above.

### MSD for Detecting Interleukin (IL)-1β, IL-6, IL-10, IL-18, and Tumor Necrosis Factor (TNF)

Serum levels of IL-1β, IL-6, IL-10, IL-18, and TNF were measured by the Meso Scale Discovery electrochemiluminescence assay (MSD, Rockville, MD, USA) according to the manufacturer’s instructions.

### Support Vector Machines

To explore whether circulating NETs signature might serve as a potential biomarker for the diagnosis of AOSD and identifying patients refractory to low-dose glucocorticoid treatment, support vector machines (SVM) approach (MATLAB R2020a) was performed. Grid search and Gaussian radial basis function kernels were implemented for tuning parameters. By using SVM, circulating NETs and clinical data of the patients in the training set were used to build a model. Then each sample in the validation set was predicted using the model and assigned a classification label. The predictive power of the model was assessed using the area under the receiver operating characteristic (ROC) curve with area under curve (AUC), sensitivity, and specificity. Overall accuracy is the ratio of correctly predicted patients.

### Statistical Analysis

Continuous variables are presented with mean ± standard deviation (SD). Categorical variables are expressed as counts and proportions. Comparisons were performed using the nonparametric Mann-Whitney U test for unpaired data, Kruskal-Wallis, followed by *post hoc* Dunn’s test for multiple comparisons. Spearman’s correlation test was used to assess the correlations. Statistical significance was analyzed by SPSS version 25.0 software (SPSS Inc., Chicago, IL, USA) and GraphPad Prism V 8.00 (GraphPad Software, San Diego, California, USA).

## Results

### Establishing Circulating NETs as a Biomarker in AOSD

**Figure 1:**
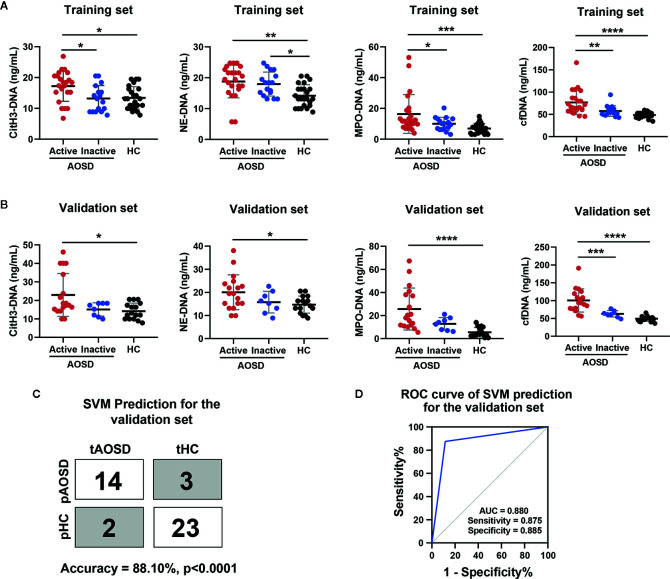
Circulating NETs signature for AOSD diagnosis in a training and validation set. **(A, B)** Comparison levels of circulating NETs (citH3-DNA, NE-DNA, MPO-DNA, and cfDNA) in AOSD patients and HCs. **(C)** SVM prediction for the validation set in distinguishing AOSD from HCs. tAOSD or tHC meant the real AOSD or HC in the validation set and pAOSD or pHC meant the predicted AOSD or HC by SVM model. **(D)** ROC curve of combined NETs signature in the validation set was analyzed by SVM analysis. AOSD, adult-onset Still’s disease; HC, healthy control; SVM, support vector machines; tAOSD, true AOSD; tHC, true HC; pAOSD, predicted AOSD; pHC, predicted HC; ROC, receiver operating characteristic; AUC, area under curve. *p < 0.05, **p < 0.01, ***p < 0.001, ****p < 0.0001.

A summary of the demographics, clinical characteristics, and laboratory findings of the study subjects can be found in **Table 1**. The training set included 40 patients with AOSD (23 active and 17 inactive) and 24 HCs. The validation set consisted of 26 patients with AOSD (18 active and 8 inactive) and 16 HCs. We then assessed circulating cfDNA and NET-DNA complexes in serum of AOSD patients and HCs, and the levels of circulating NETs in the training set are shown in **Figure 1A**. We hypothesize that NETs were associated with disease activity. All circulating NETs were higher in active AOSD than HCs (citH3-DNA: p=0.0103; NE-DNA: p=0.0017; MPO-DNA: p=0.0005; cfDNA: p<0.0001). The levels of circulating citH3-DNA, MPO-DNA, and cfDNA were significantly higher in active AOSD patients than in inactive patients (citH3-DNA: p=0.0144; MPO-DNA: p=0.0495; cfDNA: p=0.0035). There was no significant difference in the NE-DNA level between active and inactive patients with AOSD. Moreover, the levels of NE-DNA were elevated in inactive AOSD patients compared with HCs (NE-DNA: p=0.0241). In the validation set, all circulating NETs were also higher in active AOSD than HCs, similar to that in the training set (**Figure 1B**, citH3-DNA: p=0.0104; NE-DNA: p=0.0327; MPO-DNA: p<0.0001; cfDNA: p<0.0001).

**Table 1:** Clinical characteristics of AOSD patients in the training and validation set.

	Training set			Validation set		
	AOSD (n=40)			AOSD (n=26)		
	Active(n=23)	Inactive(n=17)	HC(n=24)	Active(n=18)	Inactive(n=8)	HC(n=16)
Age (Years)	35 (29,44)	41 (25.5, 54.5)	35 (29, 42)	29.5 (21.75, 53)	36.5 (27.5, 51.75)	31.5 (29.8, 34.3)
Sex (F/M)	18/5	14/3	16/8	15/3	6/2	8/8
Clinical Manifestations						
Fever	19 (82.6)	0		17 (94.4)	0	
Arthralgia	19 (82.6)	0		14 (77.8)	0	
Skin rash	20 (87.0)			14 (77.8)	0	
Sore throat	9 (39.1)	0		9 (50.0)	0	
Lymphadenopathy	11 (47.8)	1 (5.9)		15 (83.3)	0	
Splenomegaly	8 (34.8)	0		9 (50.0)	0	
Hepatomegaly	0 (0)	0		2 (11.1)	0	
Myalgia	6 (26.1)	0		6 (33.3)	0	
Pericarditis	1 (4.3)	0		4 (22.2)	0	
Pleuritis	3 (13.0)	0		5 (27.8)	0	
Pneumonia	5 (21.7)	0		7 (38.9)	0	
PAH	1 (4.3)	0		2 (11.1)	0	
Laboratory features						
Hemoglobin (g/L)	111 (104, 129)	130 (117, 138)		106 (91, 118)	126 (111, 127)	
Leukocytes (10^9^/L)	12.3 (9.0, 18.4)	8.1 (6.9, 10.1)		16.5 (11.4, 18.5)	8.5 (6.5, 10.6)	
Platelets (10^9^/L)	257 (180, 311)	178 (148, 221)		257 (164, 337)	230 (188, 290)	
ESR (mm/h)	50 (37, 71)	18 (15, 32.5)		86 (70.8, 107.5)	9.5 (8, 21.5)	
CRP (mg/L)	34.8 (21.6, 96.3)	2.1 (1.0, 11.7)		84.8 (58.9, 110.5)	2.8 (0.9, 15.5)	
ALT (U/L)	40 (27, 56)	21 (13, 32.5)		65 (20, 86)	13.5 (8.8, 31.5)	
AST (U/L)	31 (21, 52)	19 (16, 35)		43 (24.5, 85.5)	17 (14, 27.5)	
Ferritin (ng/mL)	2218 (1458, 8351)	815.3 (429.2, 1986)		10688 (5928, 15852)	1219 (634.4, 3125)	
ANA positivity	3 (13.0)	1 (5.9)		3 (16.7)	0	
RF positivity	0	0		0	0	

Data are presented as median (IQR) for continuous variables, and as frequency counts (%) for categorical variables. AOSD, adult-onset Still’s disease; HC, healthy control; PAH, pulmonary arterial hypertension; ESR, erythrocyte sedimentation rate; CRP, C-reactive protein; ALT, alanine aminotransferase; AST, aspartate aminotransferase; ANA, antinuclear antibody; RF, rheumatoid factor.

Next, we introduced combined circulating NETs to commonly used classifier SVM. The model produced by the training set exhibited excellent performance in the validation set (**Figure 1C**, accuracy = 88.10%, p<0.0001). The ROC analysis revealed that the sensitivity was 87.5%, the specificity was 88.5%, and AUC was 0.880 (**Figure 1D**). These studies suggest that the circulating NETs signature could be a potential biomarker for AOSD patients.

### Correlation of Circulating NETs With Disease Activity and Other Biomarkers of AOSD

**Figure 2:**
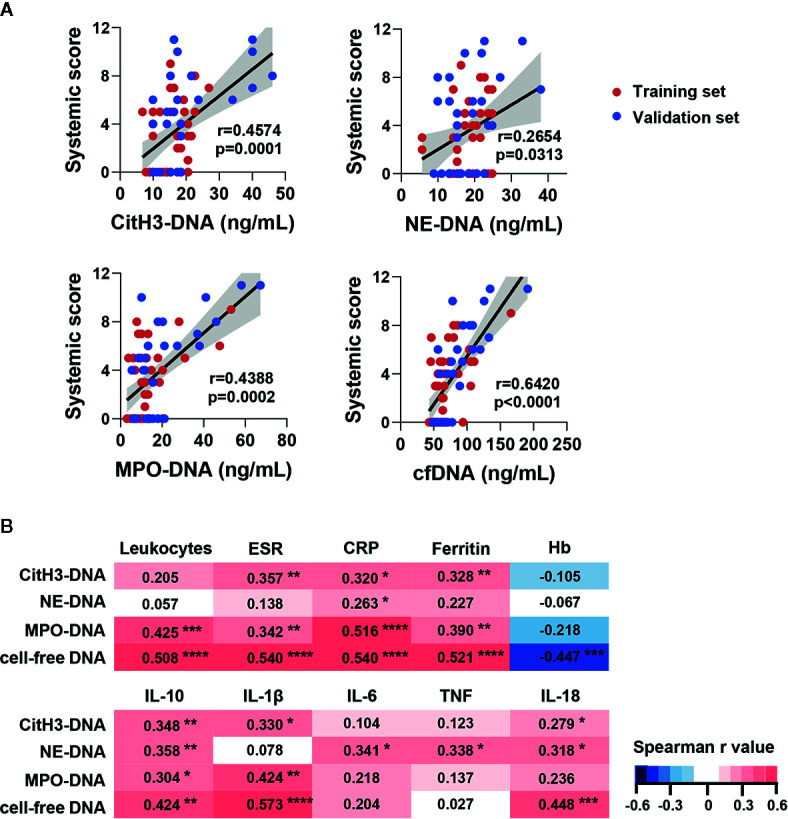
Circulating NETs signature in AOSD patients was correlated with systemic inflammation. **(A)** Circulating NETs signature was correlated with systemic score in training and validation set. **(B)** Correlation matrix of NETs signature with laboratory tests and serum cytokine level in AOSD. Heatmap manifests the strength of relationship by Spearman’s correlation analysis. AOSD, adult-onset Still’s disease; HC, healthy control; ESR, erythrocyte sedimentation rate; CRP, C-reactive protein; Hb, hemoglobin; IL, interleukin; TNF, tumor necrosis factor. *p < 0.05, **p < 0.01, ***p < 0.001, ****p < 0.0001.

To further reveal the association of circulating NETs with disease activity, we examined the correlation between NETs and AOSD systemic disease activity score. Significant correlations between systemic score and serum levels of citH3-DNA, MPO-DNA, as well as cfDNA were observed (**Figure 2A**, citH3-DNA: r=0.4574, p=0.0001; MPO-DNA: r=0.4388, p=0.0002; cfDNA: r=0.6420, p<0.0001). NE-DNA also showed a slight correlation with systemic score (**Figure 2A**, r=0.2654, p=0.0313). Taken together, our results suggest that serum levels of NETs are higher during AOSD attacks.

Next, we compared the levels of NETs in AOSD patients with routine inflammatory parameters and cytokines of AOSD. A correlation matrix were created based on the Spearman r values of the cross comparisons. The levels of citH3-DNA, MPO-DNA, and cfDNA were significantly correlated with routine inflammatory parameters. And NE-DNA only showed a slight correlation with C-reactive protein (CRP) (**Figure 2B**).

Regarding cytokines, citH3-DNA and cfDNA were correlated well with IL-1β, IL-10, and IL-18. MPO-DNA was correlated with IL-1β and IL-10 while NE-DNA was correlated with IL-10 and IL-18. Interestingly, only NE-DNA was correlated with IL-6 and TNF (**Figure 2B**). These data suggest that different types of NETs may play distinct roles and participate in diverse immune responses.

### Association Between Circulating NETs With Clinical Manifestations of AOSD

To assess the clinical potential of NETs, we analyzed levels of circulating NETs in AOSD patients with various manifestations. Patients with fever, arthralgia, skin rash, sore throat, myalgia, lymphadenopathy, hepatomegaly, splenomegaly, pericarditis, pneumonia, and pleuritis had a higher level of cfDNA than those without these symptoms (**Table 2**). Interestingly, the levels of NE-DNA were higher in patients with fever, skin rash, hepatomegaly, splenomegaly, pericarditis, pneumonia, and pleuritis (**Table 2**). The levels of citH3-DNA were increased in patients with fever, arthralgia, skin rash, myalgia, lymphadenopathy, hepatomegaly, pericarditis, pleuritic, and PAH (**Table 2**). We also observed higher levels of MPO-DNA in patients with fever, arthralgia, skin rash, myalgia, lymphadenopathy, hepatomegaly, splenomegaly, and pericarditis (**Table 2**).

**Table 2:** Comparison of the circulating NET levels according to disease manifestations in AOSD patients.

		citH3-DNA	p value	NE-DNA	p value	MPO-DNA	p value	cfDNA	p value
Fever	+, n=36	20.4 ± 9.2	0.0004	20.3 ± 5.8	0.0093	21.9 ± 16.4	0.0022	91.1 ± 31.8	<0.0001
	-, n=30	13.9 ± 4.1		16.5 ± 4.9		10.8 ± 4.5		59.2 ± 10.4	
Arthralgia	+, n=33	20.3 ± 9.7	0.0127	19.9 ± 6.3	0.0598	22.4 ± 17.0	0.0073	90.4 ± 33.8	<0.0001
	-, n=33	14.7 ± 4.2		17.2 ± 4.7		11.3 ± 4.6		62.8 ± 13.8	
Skin rash	+, n=34	20.6 ± 9.4	0.0006	20.2 ± 6.1	0.0132	21.6 ± 16.8	0.0128	89.8 ± 32.9	<0.0001
	-, n=32	14.1 ± 4.1		16.8 ± 4.7		11.7 ± 5.8		62.6 ± 15.5	
Sore throat	+, n=18	20.5 ± 9.6	0.1052	20.1 ± 7.4	0.3723	23.2 ± 19.4	0.1712	95.5 ± 39.0	0.0046
	-, n=48	16.4 ± 7.0		18.0 ± 4.8		14.4 ± 9.9		69.5 ± 20.9	
Myalgia	+, n=13	23.4 ± 8.4	0.0002	17.3 ± 5.7	0.4942	24.3 ± 16.6	0.0431	92.1 ± 30.2	0.0128
	-, n=53	16.0 ± 7.2		18.9 ± 5.7		15.0 ± 12.2		72.8 ± 27.9	
Lymphadenopathy	+, n=27	21.0 ± 10.2	0.0135	19.9 ± 6.3	0.2786	23.6 ± 17.4	0.0045	91.7 ± 36.4	0.0018
	-, n=39	15.1 ± 4.7		17.6 ± 5.1		12.2 ± 7.3		66.1 ± 16.6	
Hepatomegaly	+, n=2	40.1 ± 0.0	0.0056	35.5 ± 3.6	0.0009	47.6 ± 15.0	0.0177	133.8 ± 1.4	0.0084
	-, n=64	16.8 ± 7.0		18.0 ± 4.9		15.9 ± 12.5		74.8 ± 27.8	
Splenomegaly	+, n=17	21.3 ± 10.5	0.0792	21.5 ± 7.1	0.0290	23.9 ± 18.4	0.0439	94.2 ± 36.1	0.0038
	-, n=49	16.2 ± 6.5		17.5 ± 4.8		14.4 ± 10.6		70.5 ± 23.8	
Pericarditis	+, n=5	31.8 ± 14.3	0.0248	25.6 ± 4.5	0.0019	44.6 ± 21.8	0.0051	121.2 ± 52.1	0.0325
	-, n=61	16.3 ± 6.0		18.0 ± 5.4		14.6 ± 10.0		72.9 ± 23.7	
Pneumonia	+, n=12	21.2 ± 9.1	0.1246	22.6 ± 6.4	0.0035	21.9 ± 18.5	0.4092	96.8 ± 40.0	0.0120
	-, n=54	16.7 ± 7.5		17.6 ± 5.2		15.4 ± 12.1		71.4 ± 24.1	
Pleuritis	+, n=8	26.8 ± 13.0	0.0256	24.3 ± 4.5	0.0006	31.1 ± 24.8	0.2572	106.7 ± 45.7	0.0233
	-, n=58	16.2 ± 6.1		17.8 ± 5.4		14.9 ± 10.1		72.4 ± 23.9	
PAH	+, n=3	34.3 ± 10.1	0.0015	26.6 ± 5.8	0.0155	35.2 ± 26.5	0.3456	107.9 ± 38.9	0.1015
	-, n=63	16.7 ± 7.0		18.2 ± 5.4		16.0 ± 12.4		75.1 ± 28.2	

Levels of circulating NETs are shown as mean ± SD, and differences between two groups were analyzed using the Mann-Whitney U test for nonparametric data.

### Circulating NETs Are Associated With Liver and Cardiopulmonary System Involvement

**Figure 3:**
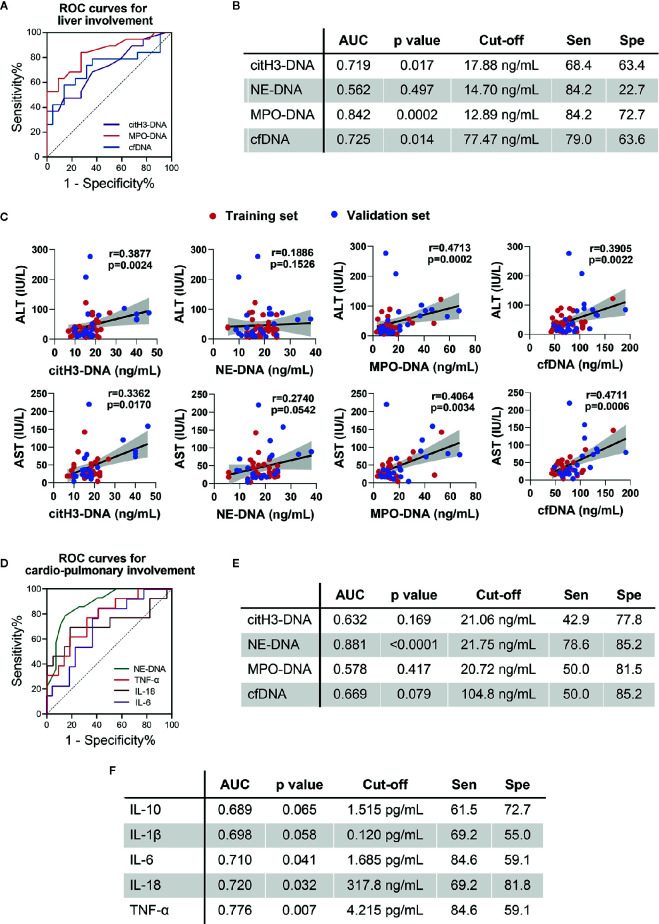
Circulating NETs and cytokines in AOSD patients with organ involvement. **(A)** ROC curve for AOSD patients with liver involvement in combined cohorts. **(B)** Sensitivity (Sen) and specificity (Spe) of circulating NETs using the cut-off determined by the ROC analysis. **(C)** Correlations of circulating NETs with ALT and AST. **(D)** ROC curve for AOSD patients with cardiopulmonary involvement in combined cohorts. **(E)** Sensitivity (Sen) and specificity (Spe) of circulating NETs using the cut-off determined by the ROC analysis. **(F)** Sensitivity (Sen) and specificity (Spe) of cytokines using the cut-off determined by the ROC analysis. AOSD, adult-onset Still’s disease; HC, healthy control; ROC, receiver operating characteristic; AUC, area under curve.

To further explore the possible clinical relevance of circulating NETs in patients with important organ involvement, we combined active AOSD from the training and validation sets and performed ROC curve analysis in patients with liver and cardiopulmonary involvements (**Figure 3A, B, D–F**). Circulating NETs performed better as compared to serum cytokines in identifying liver dysfunction in active AOSD patients. MPO-DNA could identify patients with liver dysfunction with an AUC of 0.842 (95%CI: 0.719–0.965, p=0.0002, se: 84.2%, sp: 72.7% for the value of 12.89 ng/ml). The AUC of citH3-DNA was 0.719 (95%CI: 0.561–0.877, p=0.017, se: 68.4%, sp: 63.4% for the value of 17.88 ng/ml) and the AUC of cfDNA was 0.725 (95%CI: 0.559–0.891, p=0.014, se: 79.0%, sp: 63.6% for the value of 77.47 ng/ml) (**Figure 3A, B**). The p value of ROC curves for diagnosis of liver dysfunction according to NE-DNA (**Figure 3B**) or cytokines levels (**Supplemental Figure 1**) were not statistically significant. The diagnostic value of the circulating NETs in the hepatitis of AOSD was further assessed by analyzing the correlations with gold standard liver enzymes, including ALT and AST. The circulating NETs showed strong associations with ALT and AST. In particular, ALT was correlated with citH3-DNA, MPO-DNA, and cfDNA (**Figure 3C**, citH3-DNA: r=0.3877, p=0.0024; MPO-DNA: r=0.4713, p=0.0002; cfDNA: r=0.3905, p=0.0022); AST was also correlated with citH3-DNA, MPO-DNA, and cfDNA (**Figure 3D**, citH3-DNA: r=0.3362, p=0.0170; MPO-DNA: r=0.4064, p=0.0034; cfDNA: r=0.4711, p=0.0006).

For discriminating cardiopulmonary involvement in active AOSD patients, we found NE-DNA was the sole NETs with statistical significance (**Figure 3D, E**). The AUC value of NE-DNA to identify cardiopulmonary involvement in active AOSD was 0.881 (95%CI: 0.776–0.986, p<0.0001, se: 78.6%, sp: 85.2% for the value of 21.75 ng/ml). IL-6, IL-18, and TNF could also distinguish cardiopulmonary involvement with the AUCs of 0.710, 0.720, and 0.776, respectively (**Figure 3D, F**, IL-6: 95%CI: 0.536–0.884, p=0.041, se: 84.6%, sp: 59.1% for the value of 1.685 pg/ml; IL-18: 95%CI: 0.519–0.921, p=0.032, se: 69.2%, sp: 81.8% for the value of 317.8 ng/ml; TNF: 95%CI: 0.619–0.934, p=0.007, se: 84.6%, sp: 59.1% for the value of 4.215 pg/ml). Taken together, we suspect that circulating NETs might be biomarkers of liver and cardiopulmonary involvement in AOSD.

### The Levels of Circulating NETs Predict Treatment Response to Prednisone in AOSD Patients

Among the enrolled AOSD patients, all patients received glucocorticoids with various doses. We converted different kinds of glucocorticoid into equivalent dose of prednisone and defined low-dose glucocorticoid as prednisone ≤ 1 mg/kg/day and high-dose glucocorticoid as prednisone > 1 mg/kg/day. Patients refractory to low-dose steroids were treated with high-dose glucocorticoid with or without DMARD.

**Figure 4:**
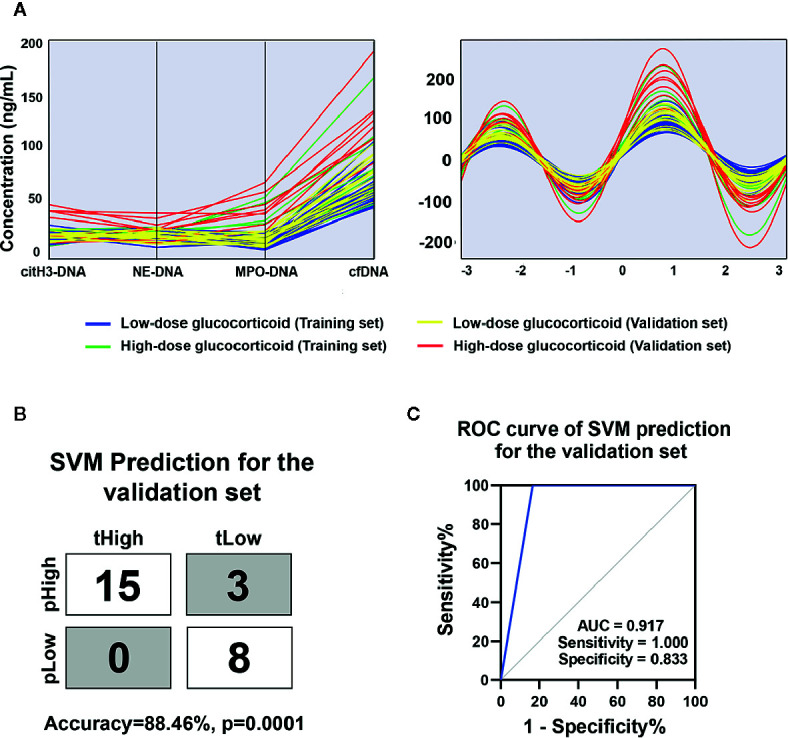
Circulating NETs signature for AOSD with different response to low-dose glucocorticoid. **(A)** Parallel coordinates plot and Andrews curve of circulating NETs in AOSD patients with different responses to low-dose glucocorticoid. **(B)** SVM prediction for the validation set in distinguishing AOSD refractory to low-dose glucocorticoid. tHigh or tLow meant the real AOSD refractory or respond to low-dose glucocorticoid and pHigh or pLow meant the predicted AOSD refractory or respond to low-dose glucocorticoid. **(C)** ROC curve of combined NETs signature in the validation set was analyzed by SVM analysis. AOSD, adult-onset Still’s disease; HC, healthy control; SVM, support vector machines; tHigh, true AOSD refractory to low-dose glucocorticoid; tLow, true AOSD respond to low-dose glucocorticoid; pHigh, predicted AOSD refractory to low-dose glucocorticoid; pLow, predicted AOSD respond to low-dose glucocorticoid; ROC, receiver operating characteristic; AUC, area under curve.

As shown in **Figure 4A**, the combined circulating NETs showed higher trends in patients refractory to low-dose steroids and could separate patients with different response to low-dose steroids by Andrew’s curves. Andrew’s curves are a method of the space transformed visualization techniques for visualizing multivariate data by mapping each observation onto a function. To further evaluate the role of circulating NETs in terms of predicting treatment response, we introduced circulating NETs to SVM. In the validation set, 23 of 26 samples were correctly classified, with an accuracy of 88.46% (**Figure 4B**, p=0.0001). We found that the sensitivity was 100%, the specificity was 83.3%, and AUC was 0.917 (**Figure 4C**). These results indicate that the circulating NETs could be potential biomarkers for predicting AOSD treatment response, and this model is capable of discriminating AOSD patients resistant to low-dose prednisone.

## Discussion

AOSD is a systemic autoinflammatory disorder of unknown etiology, commonly manifested with fever of unknown origin (1). Routine inflammatory markers, cytokines, and several molecules reflecting innate immune activation are increased in patients with AOSD and used in clinical practice (20). Our previous study demonstrated that a combined microRNAs (miRNAs) panel in plasma could be a non-invasive biomarker for the diagnosis of AOSD and to differentiate it from sepsis, providing some evidences in the study of biomarkers in AOSD (21). However, biomarkers with high specificity and sensitivity for diagnosis of AOSD organ involvement is still to be determined. Moreover, no specific biomarker enables a reliable prediction of the glucocorticoid response at the individual level (2). Inspired by recent successes in the use of machine learning in clinal practice (22), we hypothesized that machine learning applications hold promise for identifying novel disease diagnostic and prediction models in AOSD.

In the current study, we measured cfDNA and three different NET complexes in the serum of AOSD patients and made a model with multidimensional features to evaluate its clinical value. This is the first time to formulate a prediction model by machine learning for diagnosis of AOSD. We observed that significant higher levels of cfDNA, MPO-DNA, NE-DNA, and citH3-DNA were found in active AOSD patients. In addition, high NET levels were strongly correlated with routine inflammatory markers and disease activity. Thus, circulating NETs reflects systemic inflammation in AOSD patients. This hypothesis was also supported by our findings that the levels of cytokine were also closely correlated with circulating NETs. These results suggest that circulating NETs can serve as novel biomarkers for evaluating and monitoring AOSD disease activity.

Neutrophils activation has long been considered as a hallmark of AOSD pathogenesis (23). NET formation is a potent effector of activated neutrophils, with pleiotropic functions such as enhancement of immune cell recruitment, release of danger-associated molecular patterns (DAMPs), and generation of tissue damage at the site of inflammation (24). More recently, we have revealed that NETs are potential inducers of inflammasomes in macrophages, subsequently releasing inflammatory cytokines in AOSD (12). Furthermore, many studies have demonstrated that circulating NETs have great potential as novel biomarkers for autoimmune and autoinflammatory diseases (25). Much attention has been paid on the presence of NETs in AOSD patients, as well as the pro-inflammatory properties of NETs *in vitro*; however, the clinical relevance of NETs formation in AOSD has not been elucidated.

Increased level of liver enzymes, which is included in the minor criteria by Yamaguchi et al., is common in AOSD (3). Histology of AOSD liver is often characterized by mild hepatitis accompanied by neutrophilic infiltration (8). Although uninvestigated in the liver of AOSD, NETosis has been described to have a potential role in many other hepatic inflammatory conditions, i.e., by favoring the influx of monocyte-derived macrophages and enhancing production of inflammatory cytokines (26). In addition, it has been reported that NET-induced nuclear and mitochondrial damage in hepatocytes can contribute to liver injury (27). Using specific inhibitors to counteract neutrophil activation and NET release, the extent of liver injury was significantly reduced (28). Finally, it has long been suggested that NET components have cytotoxic properties that can damage endothelial cells in the liver vasculature (29). Specifically, we found that circulating NETs were correlated with liver enzymes in our AOSD patients. Emerging evidences have suggested that pro-inflammatory cytokines play pathogenic roles in mediating liver inflammation and injury (30). IL-18 has been previously reported to be associated with liver involvement of active AOSD patients (31). Of note, in this report, we demonstrated circulating NETs had better ability to evaluate liver inflammation than serum cytokines.

Cardiac and pulmonary involvements, especially pericarditis, pleural effusion, and pneumonia, are frequent in AOSD (32). Serious cardiac or pulmonary manifestations, such as myocarditis, PAH, or acute respiratory distress syndrome (ARDS), can be life-threatening in several cases (2). However, underlying pathogenesis and potential biomarkers associated with cardiopulmonary involvement in AOSD have not been investigated. The excessive infiltration of neutrophils has been recognized as pro-inflammatory and tissue-damaging in highly vascularized tissues including the heart and lungs (33). Further, it has been recognized that neutrophil-derived NETs and NETs-contained enzymes can promote epithelial and endothelial dysfunction, exacerbate inflammatory exudation, and immune cell chemotaxis (34). Here, we observed that NETs levels were closely associated with cardiopulmonary manifestations in AOSD. NE-DNA and cfDNA levels were higher in AOSD patients with pericarditis, pneumonia, and pleuritis. In particular, we found that NE-DNA level had a better performance than other NET components or cytokines by using ROC curve analysis. Numerous studies have revealed the critical role of NE in lung and heart diseases (35, 36). In addition to its proteolytic effect, NE is also known to induce pro-inflammatory cytokines in epithelial cells, thus exacerbating inflammatory damage (37, 38). Moreover, the NETs’ DNA-backbone has been demonstrated to enhance the activity of NE by protecting it from inhibition by antiproteases (39). Considering the pathogenic role of the interaction between neutrophils and endothelium in AOSD (40), NE-DNA complex may mediate vascular damage, leading to serositis and interstitial lung infiltration in AOSD. Further studies are needed to validate our findings and clarify the detailed role of NE-DNA in AOSD patients with cardiopulmonary manifestations.

Until now, there has been no standard treatment strategy for AOSD. Glucocorticoid therapy is the first line of treatment for AOSD, usually started at a dosage of 0.5–1 mg/kg/day, but higher doses of glucocorticoid may be considered if there is severe visceral involvement and/or failure to achieve remission (41). Methotrexate (MTX) is the most frequently used second-line therapy for AOSD, and hydroxychloroquine (HCQ), intravenous immunoglobulin (IVIG), and cyclosporine A (CsA) can be considered in prednisone-resistant AOSD patients (42). In recent years, cytokine inhibitors targeting IL-6 and IL-1β and Janus kinases (JAK)1/3 inhibitor tofacitinib were also found to have beneficial effects in treating refractory AOSD, though clinical evidence, especially placebo-controlled design, is limited (43). Overall, no consensus of AOSD treatment exists, and there is still a lack of reliable biomarker for predicting response to glucocorticoid therapy at the beginning. Our study suggested that circulating NETs performed well in predicting treatment response to glucocorticoid and could be novel biomarkers during first-line therapy of patients with AOSD.

Interestingly, different types of circulating NETs detected in our study had distinct abilities to identify clinical features of AOSD patients. Moreover, the correlations between different NETs and cytokines were not consistent. Recently, growing evidences have suggested that under different inflammatory context, neutrophils may express and release distinct bioactive proteins during NETosis (44). For example, NETs derived from active SLE patients were decorated with IL-17A and tissue factor (TF) (45). Conversely, neutrophils from patients with familial Mediterranean fever (FMF) and AOSD released IL-1β bearing NETs during disease attacks (46). Thus, differences in circulating NET components may be caused by distinct inflammatory microenvironment in AOSD patients. The interaction between NETosis and other inflammatory components in AOSD is a topic of critical interest for future study.

In all, we have demonstrated the close associations between increased levels of circulating NETs and organ involvement as well as glucocorticoid response in AOSD patients. Our observation is important, as it could provide information for closer monitoring disease activity or treatment of AOSD patients with organ dysfunction. It also provides a role of NETs in the pathogenesis of AOSD, suggesting that dysregulated NET formation may be involved in the liver injury and cardio-pulmonary inflammation.

## Data Availability Statement

All datasets presented in this study are included in the article/supplementary material.

## Ethics Statement 

The studies involving human participants were reviewed and approved by Institutional Research Ethics Committee of Ruijin Hospital (ID: 2016-62), Shanghai, China. The patients/participants provided their written informed consent to participate in this study.

## Author Contributions

JJ, MW, and YM: participated in NET-DNA detection, performed statistical analysis, and wrote the manuscript. JT, HS, and HL: collected the clinical samples and data. YSun and YSu: helped to revise the manuscript. JM, HC, and XChen: followed up patients. XCheng and JY: prepared the figures. TL, ZW, and LW: prepared the tables. ZZ and FW: analyzed the data of MSD. CY: designed the experiments and revised the manuscript. QH: designed experiments, performed statistical analysis, and revised the manuscript. All authors contributed to the article and approved the submitted version.

## Funding

This work was supported by Shanghai Pujiang Young Rheumatologists Training program (SPROG201901), Ruijin Youth NSFC cultivation Fund (2019QNPY01021), and National Natural Science Foundation of China (81671589, 81871272).

## Conflict of Interest

The authors declare that the research was conducted in the absence of any commercial or financial relationships that could be construed as a potential conflict of interest.

## Abbreviations

AOSD, adult-onset Still’s disease; HCs, healthy controls; MAS, macrophage activation syndrome; PAH, pulmonary arterial hypertension; DMARDs, disease modifying antirheumatic drugs; NETs, neutrophil extracellular traps; SLE, systemic lupus erythematosus; RA, rheumatoid arthritis; NLRP3, NLR family; pyrin domain containing 3; PAD4, protein-arginine deiminase 4; citH3, citrullinated histone 3; MPO, myeloperoxidase; NE, neutrophil elastase; cfDNA, cell-free DNA; RF, rheumatoid factor; ACPA, anti-citrullinated protein/peptide antibodies; dsDNA, double-stranded DNA; IL, interleukin; TNF, tumor necrosis factor; SD, standard deviation; SVM, support vector machines; ROC, receiver operating characteristic; AUC, area under curve; miRNAs, microRNAs; DAMPs, danger-associated molecular patterns; ARDS, acute respiratory distress syndrome; MTX, methotrexate; HCQ, hydroxychloroquine; IVIG, intravenous immunoglobulin; CsA, cyclosporine A; JAK, Janus kinases; TF, tissue factor; FMF, familial Mediterranean fever; ESR, erythrocyte sedimentation rate; CRP, C-reactive protein; ALT, alanine aminotransferase; AST, aspartate aminotransferase.
